# Exacerbation of branch retinal vein occlusion post SARS-CoV2 vaccination: Case reports

**DOI:** 10.1097/MD.0000000000028236

**Published:** 2021-12-17

**Authors:** Hayato Tanaka, Daisuke Nagasato, Shunsuke Nakakura, Hirotaka Tanabe, Toshihiko Nagasawa, Hiroyuki Wakuda, Yoko Imada, Yoshinori Mitamura, Hitoshi Tabuchi

**Affiliations:** a Department of Ophthalmology, Tsukazaki Hospital, Himeji, Hyogo, Japan; b Department of Technology and Design Thinking for Medicine, Hiroshima University Graduate School of Biomedical and Health Sciences, Hiroshima, Hiroshima, Japan; c Department of Ophthalmology, Institute of Biomedical Sciences, Tokushima University Graduate School, Tokushima, Tokushima, Japan; d Imada Eye Clinic, Kasai, Hyogo, Japan.

**Keywords:** adverse reaction, branch retinal vein occlusion, case report, COVID-19, vaccination

## Abstract

**Rationale:**

In this paper, we report on 2 patients who developed branch retinal vein occlusion (BRVO) exacerbation 1 day after administration of the BNT162b2 (Pfizer-BioNTech) SARS-CoV-2 vaccine.

**Patient concerns:**

Case 1: A 71 year-old female developed vision loss in her left eye 1 day after receiving a second dose of the SARS-CoV-2 mRNA vaccine. This patient was diagnosed with temporal inferior BRVO and secondary macular edema (ME) in her left eye. ME resolved after 3 doses of intravitreal aflibercept (IVA). After treatment, no recurrence of ME was observed.

Case 2: A 72 year-old man developed vision loss in his right eye 1 day after receiving the first dose of the SARS-CoV-2 mRNA vaccine. This patient was diagnosed with temporal superior BRVO in the right eye without ME. The patient was followed up and did not undergo any additional treatment.

**Diagnoses:**

Case1: Temporal superior BRVO and secondary ME were observed in the left eye. Her best-corrected visual acuity (BCVA) was 20/30.

Case2: Temporal superior BRVO recurrence and secondary ME were observed in the right eye. BCVA was 20/25.

**Interventions:**

Case1: Additional dose of IVA was administered. Case2: Two times of Intravitreal ranibizumab was administered twice.

**Outcomes:**

Case1: Subsequently, ME resolved BCVA was 20/20. Case2: Subsequently, ME resolved BCVA was 20/25.

**Lessons:**

Both cases showed a possible association between SARS-CoV-2 vaccination and the exacerbation of BRVO.

## 1 Introduction

Toward the end of 2019, China suffered from an outbreak of severe acute respiratory syndrome coronavirus 2 (SARS-CoV-2).^[1]^ Due to infection with SARS-CoV-2, acute respiratory syndrome developed in many patients. This respiratory syndrome was named coronavirus disease 2019 (COVID-19).^[2]^ SARS-CoV-2 has been rapidly spreading since its initial outbreak. With the intent of limiting the further spread of SARS-CoV-2, vaccination campaigns have been ongoing all over the world.^[3]^ Common reactions after vaccination include pain, redness, swelling around the injection site, fever, headache, myalgia, and fatigue.^[3]^ Anaphylaxis has also been reported as a relatively rare but severe reaction.^[4]^ Thrombosis, hemorrhage,^[5–7]^ and myocarditis^[8]^ after vaccination have also been identified. Adverse events affecting the eyes after vaccination have also been reported. These include keratoplasty rejection,^[9,10]^ panuveitis,^[11]^ and abducens nerve palsy^[12]^; however, the association between SARS-CoV-2 vaccination and retinal vascular occlusion disease remains unclear.

In this paper, we report 2 cases of individuals being treated or followed with branch retinal vein occlusion (BRVO), who developed a recurrence of BRVO 1 day following SARS-CoV-2 vaccination with BNT162b2 by Pfizer-BioNTech.

## 2 Case report

### 2.1 Case 1

**Figure 1:**
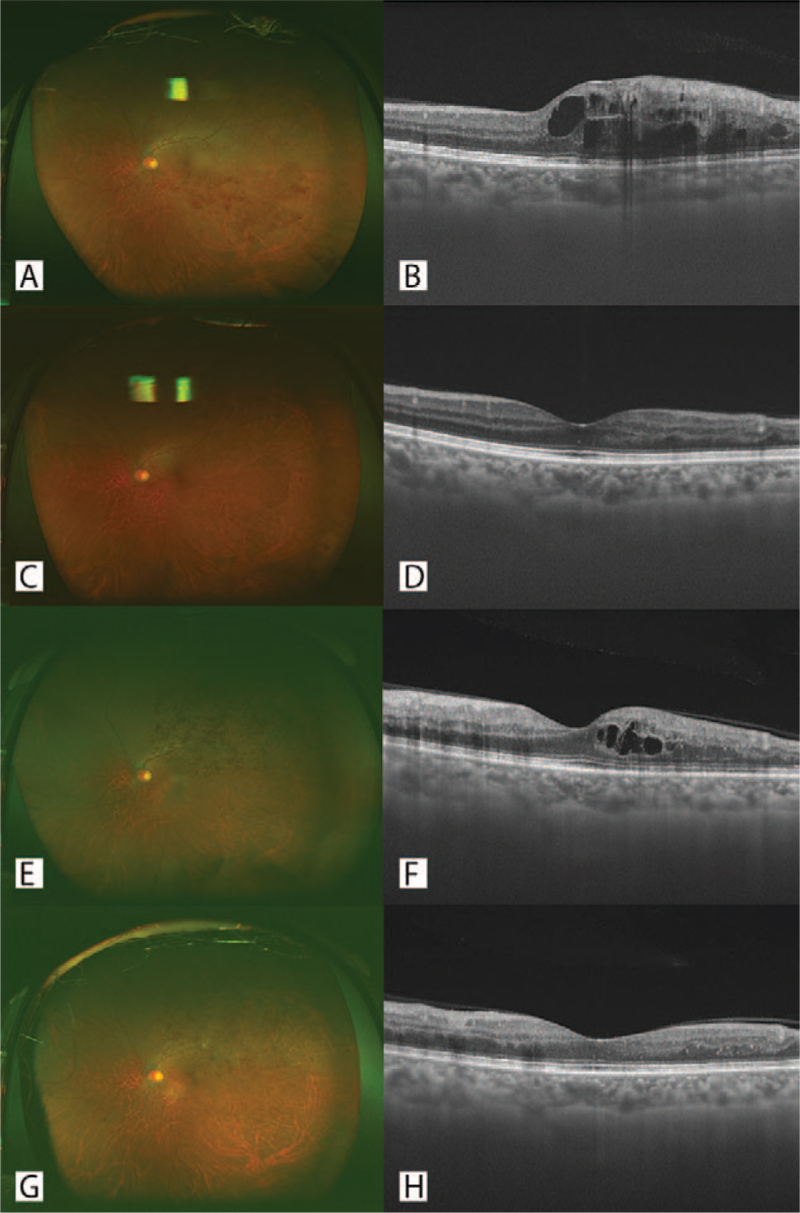
Ultra-wide-field pseudo-color (UWPC) and sagittal optical coherence tomography (OCT) images of the left eye of case 1 patient. (A, B) UWPC and sagittal OCT images at initial presentation. Temporal inferior branch retinal vein occlusion (BRVO) (A) and macular edema (ME) in fovea (B) were shown. (C, D) UWPC and sagittal OCT images after 3 times of intravitreal aflibercept (IVA). ME disappeared (D). (E, F) UWPC and sagittal OCT images after BNT162b2 vaccination. Temporal superior BRVO (E) and recurrence of ME (F) were shown. (G, H) UWPC and sagittal OCT images after additional IVA. ME disappeared again (H).

A 71-year-old woman visited our hospital in January 2018 with vision loss affecting her left eye. The best-corrected visual acuity (BCVA) in the left eye was 13/20. Ultra-wide-field pseudo-color (UWPC) and optical coherence tomography (OCT) images showed inferior temporal BRVO and secondary macular edema (ME) in her left eye. (Fig. 1A, B) Her left eye received 3 doses of intravitreal aflibercept (IVA), which resolved her ME. (Fig. 1C, D) Her BCVA was 20/20. No recurrence was found at the time of follow-up. This patient had not previously been infected with SARS-CoV-2. She received a second dose of the SARS-CoV-2 mRNA vaccine BNT162b2 (Pfizer-BioNTech) in July 2021. The following day, the patient noticed vision loss in her left eye. Her BCVA decreased to 20/30, and her UWPC and OCT images showed superior temporal BRVO and secondary ME in her left eye. (Fig. 1E, F) No avascular area was found on OCT angiography. The left eye received an additional one-time dose of IVA. The ME resolved (Fig. 1G, H), and the BCVA improved to 20/20.

### 2.2 Case 2

**Figure 2:**
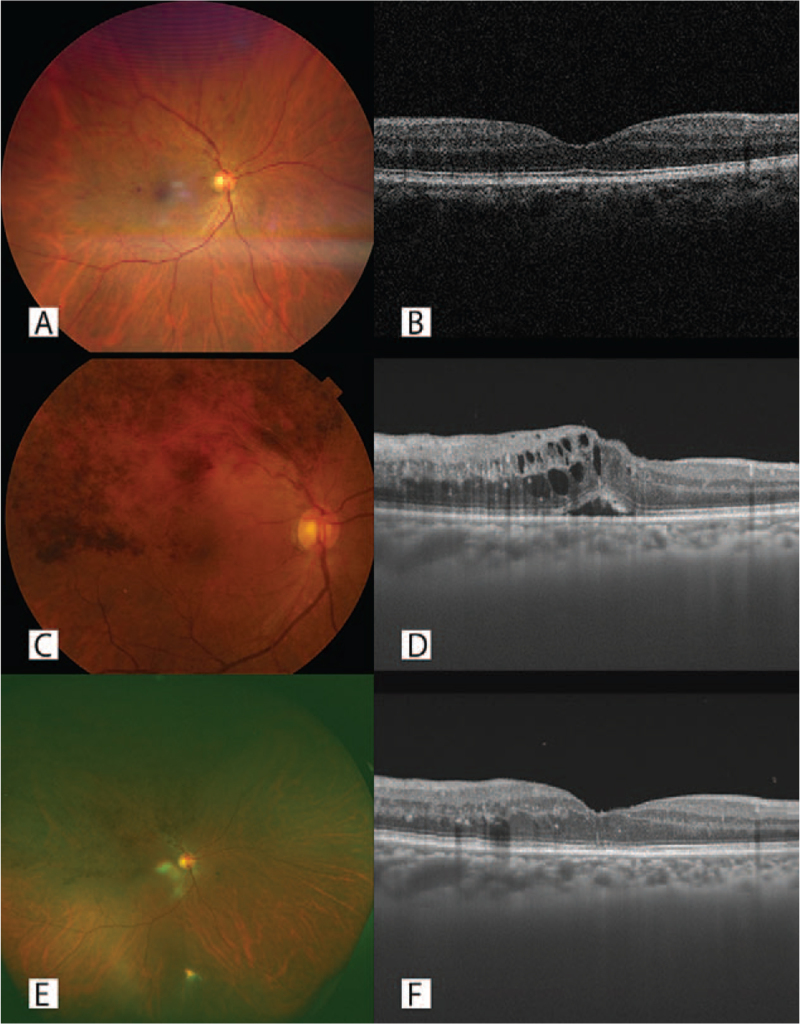
Posterior pole fundus photographs, ultra-wide-field pseudo-color (UWPC) image, and sagittal optical coherence tomography (OCT) image of the right eye of case 2 patient. (A, B) Posterior pole fundus photograph and sagittal OCT image at initial presentation. Temporal superior branch retinal vein occlusion (BRVO) (A) was shown, but macular edema (ME) was not found in fovea (B). (C, D) Posterior pole fundus photograph and sagittal OCT image after BNT162b2 vaccination. Exacerbation of temporal superior BRVO (C) was shown, and ME was found in fovea (D). (E, F) UWPC and sagittal OCT images after 2 times of intravitreal ranibizumab. ME disappeared (F).

A 74 year-old man visited our clinic in February 2020 with a complaint of vision loss affecting the right eye. His BCVA in the right eye was 20/20. His posterior pole fundus photographs and OCT images showed temporal superior BRVO without ME. (Fig. 2A, B) He was followed without treatment. He did not have a prior SARS-CoV-2 infection. He received his first dose of the SARS-CoV-2 mRNA vaccine BNT162b2 (Pfizer-BioNTech) in July 2021. On the following day, the patient noticed vision loss in his right eye. The patient was referred to our hospital. His UWPC and OCT images showed recurrence of temporal superior BRVO and secondary ME. (Fig. 2C, D) His BCVA was 20/25. No avascular area was found on the Optical coherence tomography angiography images. His right eye received 2 doses of intravitreal ranibizumab, which resolved the ME. His BCVA was 20/25. (Fig. 2E, F) The patient received his second vaccination dose 3 weeks after his first. He did not have any symptoms, nor did he have any changes in the examination of his right eye after receiving this second dose.

## 3 Discussion

Several cases of retinal vascular disease development after COVID-19 diagnosis were reported in 2020. Alessandro et al^[13]^ reported a case of central retinal vein occlusion 2 days after the COVID-19 diagnosis. Neslihan et al^[14]^ reported a case of central retinal artery occlusion (CRAO) and paracentral acute middle maculopathy 2 weeks after COVID-19 diagnosis. Andrea et al^[15]^ reported a case of Central retinal vein occlusion after hospitalization for COVID-19 pneumonia. Systemic inflammation to fight against SARS-CoV-2 infection is reported to damage vessel endothelium and bring about coagulative dysfunction, which may subsequently trigger thrombosis.^[16]^ These reports indicated that vessel endothelial damage and coagulative dysfunction caused by COVID-19 can result in CRVO and CRAO.

Our report showed an association between SARS-CoV-2 vaccination and BRVO. SARS-CoV-2 vaccination initiates a systemic inflammatory reaction, which imitates the immunogenic reaction observed in patients with COVID-19. For example, in the body of BNT162b2 recipients, cells take up mRNA in lipid nanoparticles and translate the mRNA into the spike protein of SARS-CoV-2. The cells then display spike protein on their surface, which is followed by a downstream immune response, ultimately leading to immunization against SARS-CoV-2.^[17]^ The process of the inflammatory reaction after SARS-CoV-2 vaccination is the cause of pain, redness, swelling around the injection site, fever, headache, myalgia, and fatigue, which Polack et al^[3]^ reported. Therefore, it is hypothesized that the same immunogenic reaction in SARS-CoV-2 vaccination recipients can affect the vessel endothelium, resulting in dysregulated coagulation leading to thrombosis in vessels of the retinal vascular system.

Several reports confirming the risk of vascular disease after SARS-CoV2 vaccination have been published since early 2021. Smadja et al^[18]^ reported that VigiBase rates for venous and arterial thrombotic events—lower limb thrombosis, pulmonary embolism, stroke, myocardial infarction, and so on, were seen in cases after BNT162b2, mRNA-1273, and AZD1222 vaccination. Since the establishment of the WHO Program for International Drug Monitoring in 1968, vigibase is the world's largest pharmacovigilance database, with submissions from member states. Anton et al^[5]^ reported that the 28-day rates of hospital contacts for venous thromboembolism and bleeding among people after vaccination with Oxford-AstraZeneca AZD1222 were higher than expected. See et al^[6]^ reported 12 cases of cerebral venous sinus thrombosis with thrombocytopenia within 15 days after Johnson and Johnson Ad26.COV2.S vaccination. These 2 reports suggest that the suggested potential mechanism is related to platelet-activating antibodies, which they referred to as vaccine-induced thrombotic thrombocytopenia. BNT162b2 may have a different mechanism of thrombosis with adenovirus vector vaccines, including AZD1222 and Ad26.COV2.S.

The association between SARS-CoV-2 vaccination and retinal vascular disease exacerbation in cases 1 and 2 cannot be asserted; however, we assume that vaccination was the trigger for BRVO recurrence. Case 1 developed a BRVO with ME 3 years ago, had an improvement in her ME following IVA and did not show recurrence of ME, which required additional treatment. Case 2 developed a BRVO without ME 1 year ago, and another ME had not appeared since that time. Both cases experienced recurrence and exacerbation of BRVO the day after administration of the BNT162b2 vaccine. Based on the findings in these 2 cases, we hypothesize that SARS-CoV2 vaccination can bring about a systemic immunogenic response and secondary coagulative dysfunction similar to that of COVID-19. This coagulative dysfunction can result in BRVO recurrence.

## 4 Conclusion

We report 2 cases of BRVO recurrence the day after SARS-CoV-2 vaccination. Medical workers promoting SARS-CoV-2 vaccination should be made aware that SARS-CoV-2 vaccination poses a potential risk of retinal vascular occlusion exacerbation. We suggest that more studies be conducted to determine whether BRVO exacerbation leading to macular edema should be categorized as a potential risk of this vaccine.

## Acknowledgments

We thank the ophthalmology staff of Tsukazaki Hospital who were involved in the care of these patients.

## Author contributions

**Conceptualization:** Daisuke Nagasato, Hiroyuki Wakuda.

**Investigation:** Daisuke Nagasato.

**Project administration:** Toshihiko Nagasawa, Hitoshi Tabuchi.

**Resources:** Hirotaka Tanabe, Yoko Imada.

**Supervision:** Shunsuke Nakakura.

**Writing – original draft:** Hayato Tanaka, Daisuke Nagasato.

**Writing – review & editing:** Daisuke Nagasato, Yoshinori Mitamura.
